# The Problem of Home Therapy during COVID-19 Pandemic in Italy: Government Guidelines versus Freedom of Cure?

**DOI:** 10.26502/fjppr.055

**Published:** 2022-08-02

**Authors:** Serafino Fazio, Marco Cosentino, Franca Marino, Sergio Pandolfi, Elisabetta Zanolin, Paolo Bellavite

**Affiliations:** 1 Medical School University Federico II, Naples (retired professor), Italy; 2 Center of Research in Medical Pharmacology, University of Insubria, Varese, Italy; 3 High School Master of Oxygen-Ozone Therapy, University of Pavia, Italy; 4 Department of Diagnostics and Public Health, University of Verona, Italy; 5 Medical School University of Verona, Verona (retired professor), Italy

**Keywords:** COVID-19 treatment, treatment guidelines, COVID-19 lethality, outpatient care, SARS-CoV-2 epidemiology, paracetamol, healthcare system

## Abstract

After starting in late 2019, COVID-19 spread worldwide, and Italy was one of the first Western nations to be seriously affected. At that time, both the virus and the disease were little known and there were no Evidence-Based Medicine indications for treatment. The Italian Health Ministry guidelines claimed that, unless oxygen saturation fell to <92%, no pharmacological treatment was necessary during the first 72 hours, other than on a purely symptomatic basis, preferably with paracetamol. As later confirmed, that delay in therapeutic intervention may have been responsible for numerous hospital admissions and a very high lethality (3.5 %). To try to remedy this situation, several volunteer groups were formed, managing to promptlycure thousands of patients at home with non-steroidal anti-inflammatory drugs and a variety of re-purposed drugs (principally hydroxychloroquine, ivermectin) and supplements (such as antioxidants, polyphenols and vitamin D). Although not documented by any randomized controlled studies, these approaches were nonetheless based on the best available evidence, were aimed at addressing otherwise unmet major needs and produced a significant reduction of hospitalizations, of symptom duration, and a complete recovery from the disease compared with late treatment, according to some retrospective observational studies and the clinical experience of many physicians. A prompt discussion, with a clear and open exchange between healthcare Institutions and the said groups of voluntary physicians, could clarify the most effective approaches to reduce the number of hospitalizations and the lethality of this disease.

## 1. Introduction

COVID-19 is a new disease due to the acute severe respiratory syndrome corona-virus-2 (SARS-CoV-2). After its onset in Wuhan, China, at the end of 2019, it rapidly spread worldwide leading to the development of a pandemic. After China, Italy was the first Western nation to be severely affected and, during the first months of 2020, the health system care was particularly disoriented and unprepared to deal with the surging epidemic. In this paper we analyze in historical and narrative terms the evolution of the therapeutic approaches to COVID-19 in Italy and the critical discussions that arose between doctors and public health professionals working within the communities and the government and regulatory authorities.

Since the 1990s, the practice of medicine has been completely changed by the advent of evidence-based medicine (EBM), leading to major changes and advances in the medical management of patients. The scientific demonstration of previously established evidence seeks to prevent errors as far as possible. It is therefore obvious that faced with established medical evidence it would be truly inconsistent not to use it. However, it should also be kept in mind that, at the onset of the SARS-CoV-2 pandemic, when the virus and ensuing disease were scarcely known, there were no EBM-based guidelines for the proper treatment of this disease available, nor would there be for a long time to come. Furthermore, the vast majority of doctors who found themselves dealing with the new pandemic situation had grown up and practiced professionally in an EBM environment and many of them felt ill at ease to treat patients with COVID-19 at home, lacking the support of clear guidelines.

For all these reasons, it would have been absolutely necessary for the competent international institutions to step in, such as the World Health Organisation, the major regulatory bodies, such as the Food and Drug Administration and the European Medicine Agency, and in the case of Italy, in particular, the Ministry of Health and the Italian Medicines Agency (AIFA), to establish that – lacking any available medical evidence – it was up to the medical judgment of each doctor to decide the best possible treatment, on a case by case basis, in agreement with the generally accepted ethical principles of the medical profession (cf. Article 13 of the code of ethics of Italian doctors at https://portale.fnomceo.it/codice-deontologico/) and with the applicable laws (in Italy, prescription outside authorized indications is regulated by Law 94 of 1998).

Already at the beginning of 2020, some of us were expressing an opinion on the subject by saying that “*while waiting for definite EBM-based facts for the treatment of COVID-19, it would be absolutely unethical to leave patients at home without treatment, ac-cepting the risk that the disease could worsen, instead we should be looking to try other, even off-label, pharmacological treatments with available well-proven drugs for other conditions, which might prove effective against COVID-19 as well, based on the pathophysiological mechanisms of the disease, which were gradually becoming known and on the well-known actions of these drugs*” [1]. Even back then, Indomethacin was already indicated as one of the drugs that could bring benefits in the treatment of COVID-19, due to both its powerful anti-inflammatory action and, above all, its known antiviral action [2].

In Italy, instead, the Ministry of Health issued recommendations to treat the disease on a symptomatic basis alone, using only paracetamol for fever and pain, waiting and monitoring oxygen saturation by means of a pulse oximeter and intervening only in the event that the oxygen saturation level dropped below 92%, when hospitalization would be inevitable [3]. These guidelines failed to take into account the fact that the disease has several stages and in many patients, if not adequately treated, the stage of respiratory tract infection is rapidly and umpredictably followed by severe multi-systemic inflammation with involvement of the coagulation systems and cytokine and bradykinin “storms”. Moreover, a drop in oxygen saturation to below 92% indicates that significant damage has by then already occurred to the lungs, and probably to other organs as well, and that the risk of other chronic conditions setting in could not be ruled out, even after recovery from COVID-19, with a subsequent burden on the national health system.

These official recommendations also stated that no drugs were needed before 72 hours (pages 9 and 10) [3]. In the presence of a diagnosed infectious disease, however, it seems unethical to stand by and not initiate pharmacological treatment to combat the infection and attempt to halt progression of the disease, particularly in this case where it has been seen that the subsequent stages of the disease can be truly dangerous to the point of the possible onset of complications which then becomes difficult to control. This delay in home care, as subsequently confirmed, may have been responsible, particularly in the first months of the year 2020, for both the high number of hospital admissions, with overloading of hospitals that were ill-organised to respond to the rising demand, and the very high lethality rate recorded at the time (13.7%).

Worldwide, but particularly in Italy, the pandemic has been tackled almost exclusively through anti-COVID-19 vaccination, even though these vaccines were still only authorized for emergency use and therefore their safety, in the short, medium and long term, had not yet been clarified with certainty. Furthermore, it became apparent as the months went by that not only did the vaccine not prevent the possibility of being infected with the virus, as a result of which even vaccinated persons could spread the infection, but also that the vaccine rapidly reduced its level of protection, thus requiring boosters, which were also administered with different vaccines from the basal vaccine. Thus, even many fully vaccinated persons caught the disease, albeit mostly less severely, although a number of them still required hospitalization, intensive care, and many even died [4–6]. A discussion of the worrying signals of the pathogenicity of these newly designed vaccines and of the variable benefit / risk ratio in different ages of life is beyond the scope of this paper.

In the light of these facts, which are becoming more and more obvious as experiences of physicians around the world are gathered, it is necessary to ask whether focusing all resources on the vaccine was a good choice. So why not also focus on home therapy protocols, even while waiting for precise EBM-based treatment protocols? In Italy, many doctors have treated patients at home, also by way of telemedicine systems, drawing on their knowledge and guided by science and conscience. The majority of these doctors, seeing that the disease triggered strong organ and systemic inflammation, associated with episodes of micro- and macro-thrombosis, used NSAIDs in association with antithrombotic drugs (antiplatelet or low molecular weight heparins), plus gastric protection.

The poor response and organisation of local community level healthcare in Italy, in the face of the pandemic, led to the formation of several groups of volunteers, mainly doctors but also other healthcare and non-healthcare professionals, who joined forces in an attempt to remedy the situation that had been created and help healthcare facilities in the battle against the pandemic, providing great support to the disoriented and frightened public. The largest among the many groups set up at the time were the “*Early Home Therapy for COVID-19*” group (https://www.terapiadomiciliarecovid19.org), founded in March 2020 by a lawyer, Erich Grimaldi, and the “Ippocrate.org” group (https://ippocrateorg.org/), founded between May and July 2020 by Mauro Rango.

Both organisations have since treated thousands of cases of COVID-19 at home, also by remote monitoring, using personalized treatments with drugs, mainly non-steroidal anti-inflammatory drugs (NSAIDs) but also dietary supplements and repurposed drugs, administered, when possible, at the onset of the symptoms, in order to prevent clinical worsening and promote a full recovery. This type of approach, according to all participating physicians, based on clinical observation, has led to excellent results, as documented by the clear drop in hospitalizations, the duration of symptoms and the number of deaths. Preliminary results with retrospective case histories of these experiences have already been published by our research groups [7, 8].

## 2 Lethality trend in Italy during the pandemic: Determining factors

When SARS-CoV-2 first appeared, COVID-19 was a virtually alien disease, the pathophysiological mechanisms of which were unknown, and which could even lead to the death of the infected person. Therefore, all useful measures had to be taken and implemented to clarify the relevant mechanisms and to investigate the nature of this disease as quickly as possible, in order to be able to treat it in the best possible way. To this end, autopsies of COVID-19 patients are certainly the quickest and most important of the various means at our disposal to understand the mechanisms and effects of the disease on our bodies, yet the circular of the Italian Ministry of Health of 1 April 2020, no. 11285 [9] (Page 3), reads as follows on the subject of autopsy examinations and diagnostic findings on deceased COVID-19 patients: “*For the entire period of the emergency phase, no autopsies or diagnostic findings should be carried out in con-firmed cases of COVID-19...*”.

While not a blanket ban, this led to no or very few autopsies actually being carried out, precisely during the period when they would have been crucial to rapidly further our knowledge of the disease. In fact, it was precisely thanks to subsequent studies, carried out using the autopsy method, that it became clear that one of the factors that aggravated the disease was endothelitis with the triggering of pulmonary micro- and macro-thromboses, as well as multi-district thrombosis [10]. It was precisely for this reason that a new circular of the Italian Ministry of Health, dated 11/01/2021, no.0000818 [11], corrected the aim by removing the wording that “advised against” performing autopsies.

Factors such as the significant impact of COVID-19 on the elderly population, the decision to manage the disease at an advanced stage only and exclusively in hospital settings, and the critical nature of the home treatment approach to people infected with SARS-CoV-2, may have contributed to the increase in the number of deaths from COVID-19 in Italy [12, 13]. The average lethality rate for COVID-19 in Italy was very high in 2020 (3.5%) and has gradually declined to 0.33% today, with an average figure for the entire pandemic period of about 1% (Table 1, data from Worldometers Coronavirus site). Vice versa, in countries such as Portugal (Table 1), where the healthcare service responded particularly well, due to an excellent organisation at local community level, the average recorded lethality during the pandemic was 0.55%, i.e., still about 50% lower than in Italy [12], and this cannot be justified only by the fact that the over-65 segment of the population in Italy is 21%, while in Portugal it is 18%.

**Figure 1:**
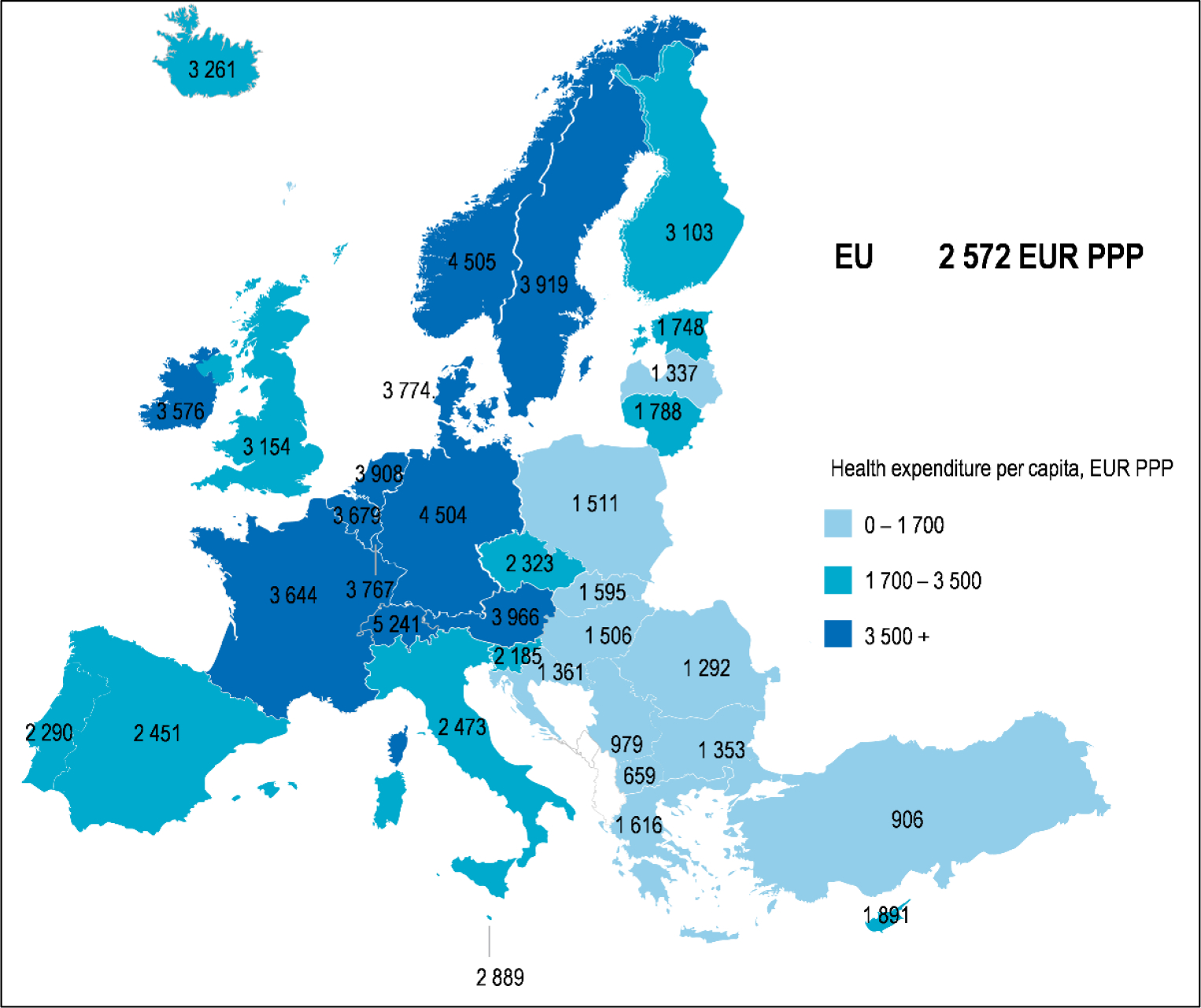
Per capita health expenditure in Europe, 2019 (From OECD iLibrary.org, Health at a Glance, Europe 2020)

Among the Western European countries, Italy recorded the highest lethality rate: in fact, Spain and Greece had an average lethality of 0.86%, Belgium 0.76%, Germany 0.53%, France 0.50%, Austria 0.44%, Luxembourg 0.43%, Switzerland 0.38%, and the Nether-lands 0.27%. Even Sweden, accused of pandemic mismanagement for not applying strict restrictions during the various pandemic waves, recorded a lethality of 0.75%. On the other hand, according to the latest OECD iLibrary.org report, Italy is one of the tail-end countries in terms of per capita health expenditure, comparable to Greece, Spain and Portugal, which, however, recorded a much lower average lethality rate. (From OECD iLibrary.org, Health at a Glance: Europe 2020: State of Health in the EU Cycle) (Figure 1).

There are many likely causes of the important and progressive reduction in apparent lethality of COVID-19, from the beginning of the pandemic to the present day, although the most relevant seem to us to be the following:

1. NATURAL SELECTION. A relevant factor was the gradual reduction in the number of frail elderly people, who died in considerable numbers during the first months of the pandemic, with the average age of the sick decreasing and thus the number of cases destined to unfavorable evolution.
2. THE VACCINATION CAMPAIGN. The vaccination campaign, while failing to reduce the number of infections, probably conferred some degree of protection from serious illness and death for COVID-19 in elderly and more frail patients, thereby reducing lethality. However, in actual fact, the authorization studies on COVID-19 vaccines have not provided any evidence of a possible reduction in deaths from COVID-19 [14] since all-cause mortality was not substantially different over a six month period comparing subjects in whom was administered the BNT162b2 vaccine (15 deaths) with those who received placebo (14 deaths), and that there were more cardiovascular and sepsis related deaths in the first group (12 deaths) that in the second (6 deaths). However, a booster dose at least 5 months after a second dose of BNT162b2 added protection also against mortality [15], thus showing that, perhaps, the protection given by vaccination in the short term for deaths by COVID-19 may be lost in the long term as number of all-cause mortalities, in the absence of subsequent booster. It should be noted that the effect of anti-covid-19 vaccines on public health should also be evaluated in the light of the emerging evidence of the multiple adverse effects caused by them, which are not always reported.
3. THE GRADUAL INCREASE IN THE NUMBER OF SWAB TESTS. Swab testing became mandatory to access the workplace, public services and all indoor premises, such as restaurants, cinemas, theatres, etc., initially for those persons who did not wish to be vaccinated against COVID-19, but which was then extended also to vaccinated persons after it was realized that the COVID-19 jab did not provide protection against the spread of the virus. In order to be able to have a working and social life, therefore, it was necessary to either complete the vaccination program or to have been cured of COVID-19 within the last 6 months or to have negatively tested for SARS-CoV-2 every 3 days. Performing these blanket tests in the population, in order to go about one’s working and social life, resulted in the discovery of asymptomatic positives. It has been speculated that the number of asymptomatic positives among the population with confirmed COVID-19 may be highly variable, but averages around 40.5% of population [16]. The lethality then progressively decreased because so many patients were asymptomatic, particularly during the wave of the omicron variant.
4. NEW THERAPEUTIC APPROACHES. A fourth quite relevant factor was the progressive improvement of the health response in dealing with the pandemic, with the discovery of the efficacy of certain drugs (in particular, NSAIDs but also monoclonal antibodies and appropriate use of steroids and antivirals) in preventing disease progression and the realization of the need for early intervention by doctors, even in the absence of official guidelines [7, 8, 17–22].

### LESS AGGRESSIVE VARIANTS.

Last but not least, one has to consider the advent of the Omicron variant, which is highly infectious but with a markedly diminished virulence compared to the previous variants. This, too, has led to an exponential increase in cases, but fortunately with fewer deaths, further contributing to the drop in lethality which, referring to data from the Worldometers Coronavirus website, has fallen from 3.5% in 2020 to 1.55% in 2021 and 0.33% in the first months of 2022 (up to 18 May), settling at an average lethality of 1% over the entire pan-demic period.

## 3. Reports from the Ministry of Health and responses from doctors who acted in the area

During the pandemic a clear dichotomy arose between local community doctors and emergency room and hospital doctors, the former coming into contact with the sick at an early stage of the infection and becoming accustomed to treating the disease at the onset of the first symptoms to prevent it from worsening, while the latter doctors almost exclusively treating the sick in the later/worse stages (second and third phases) of the disease, which had to be treated promptly according to guidelines already available for hospitalized patients. These are very different stages of the disease that require completely different approaches. Furthermore, while there are still no randomized and controlled trials that have produced guidelines for the early home treatment of patients with mild and/or moderate low-risk COVID-19, there are recommendations for early treatment, based on randomized controlled trials, for patients at risk of worsening with monoclonal antibodies [19, 22] and the new antiviral drugs remdesivir, molnupinavir, ritonavir plus nirmatrelvir [20, 21]. Unfortunately, however, the pandemic has shown us that even patients with mild and/or moderate COVID-19, if left untreated, can deteriorate, require hospitalization and eventually die [23].

Therefore, close cooperation between the various health care components and institutions would have been necessary to fully exchange information and collaboration, which could have resulted in better controlling the pandemic. Despite urgent and re-peated requests, the Minister of Health never accepted to meet with the representatives of the doctors who voluntarily treated patients at home at the first symptoms of the disease, free of charge, thus building up a great deal of experience on the disease, in order to discuss the matter. Groups of doctors have called for changes to be made to the guidelines, even resorting to the courts, but the Ministry of Health has consistently opposed these requests. A history of the legislative clashes between doctors and the Ministry of Health and AIFA can be found in a Journal of Administrative Law [24] and is summarized below (see also Table 2).

The guidelines of the Italian Ministry of Health (henceforth the “Ministry”) echoed those of the Italian Medicines Agency (AIFA) based on three distinct stages of the disease:

1. An early stage, clinically characterized by the appearance of general malaise, fever and a dry cough. Cases in which the host’s immune system succeeds in blocking the infection at this stage (the majority) have an entirely benign course.
2. The disease may then evolve into a second stage featuring morpho-functional alterations in the lungs caused by both the cytopathic effects of the virus and the host’s immune response. This phase is characterized by interstitial pneumonia, very often bilateral in nature, associated with respiratory symptoms that are generally limited in the early phase, but which may subsequently lead to progressive clinical instability with respiratory insufficiency.
3. The second scenario, in a limited number of people, can evolve towards a clinical picture dominated by the cytokine storm and consequent hyperinflammatory stage, which leads to local and systemic consequences and represents a negative prognostic factor producing, in the lungs, arterial and venous vasculopathy with blood clots forming in the small vessels and evolution towards serious and some-times permanent pulmonary lesions (pulmonary fibrosis).

This classification was correct and in agreement with the main scientific reference literature (e.g: [27, 28], but was followed by the very questionable definition of “low-risk” patient in this way (quoted literally with our translation):

- absence of increased risk factors (e.g., neoplastic diseases or immunodepression)
- flu-like symptoms (e.g., rhinitis, cough without difficulty breathing, myalgia, headache);
- absence of dyspnea and tachypnoea (documenting SpO2 > 92% whenever possible);
- fever ≤38 °C or >38 °C since less than 72 hours;
- gastro-enteric symptoms (in the absence of dehydration and/or multiple diarrheal discharges);
- asthenia, ageusia / dysgeusia / anosmia.

**Figure 2:**
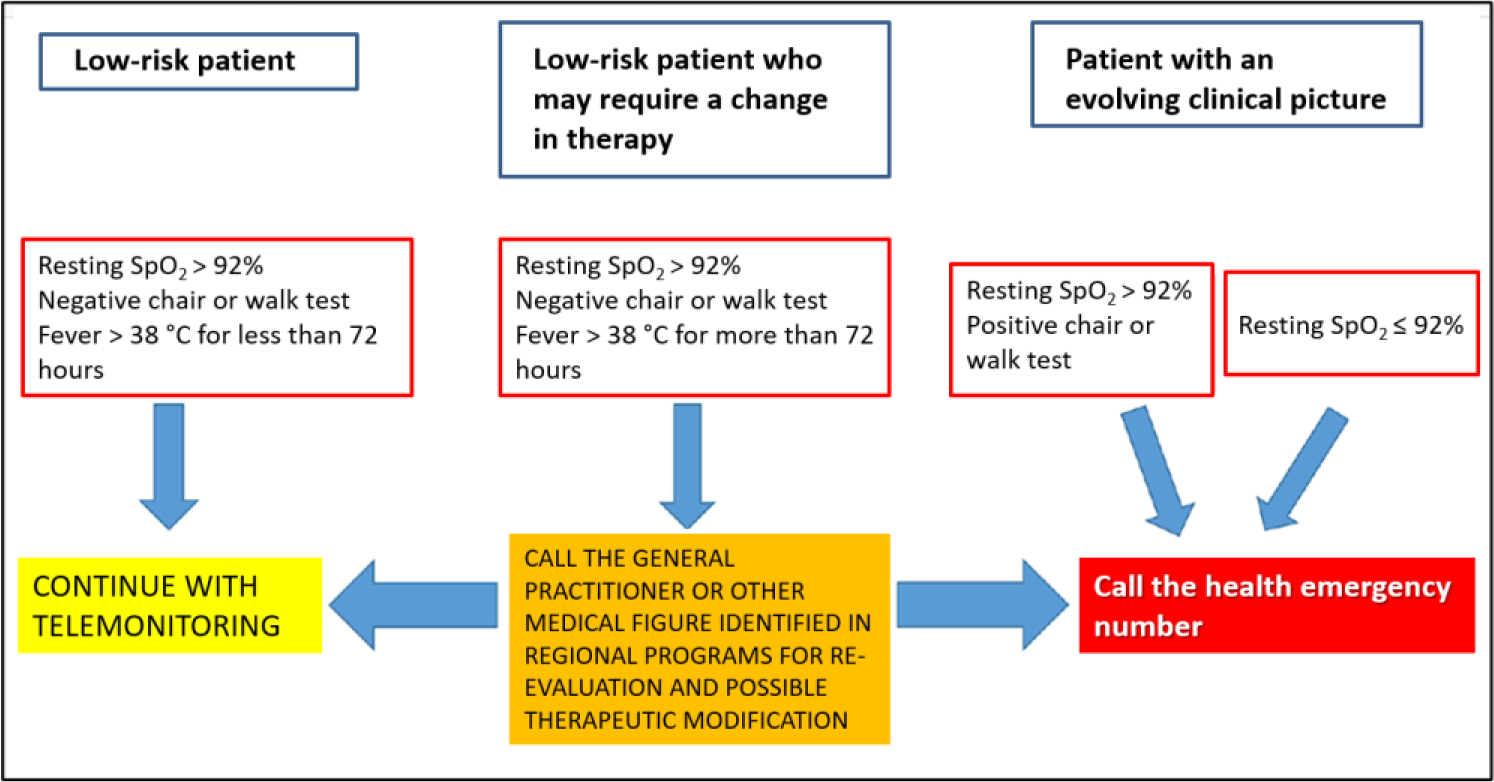
Outline of COVID-19 home treatment according to the Italian Health Ministry guidelines (see text).

In other words, patients with overt COVID-19 symptoms were judged to be at low risk and to only require “remote monitoring”, as a recommendation, according to an algorithm shown in Figure 2.

The strategy shown here, which is still in force in the Ministry’s guidelines, is highly objectionable, first and foremost because it considers patients with overt COVID-19 to be at “low risk” for the first 72 hours and only recommends “*remote monitoring*” for these patients. In fact, for low-risk patients, the advisable treatment is expressed in these words (our translation): “*Generally speaking, for subjects with these clinical features, no treatment is recommended except possible for a symptomatic supportive therapy*”. In particular, in asymptomatic or paucisymptomatic subjects at home, a so called “watchful waiting”, including periodic measurement of oxygen saturation levels by means of pulse oximetry, symptomatic treatment (e.g., paracetamol) and appropriate hydration and nutrition were recommended, as well as specific indications for im-munocompromised patients [3].

Furthermore, the home treatment guidelines recommended not to routinely use corticosteroids, not to use heparin, except in immobilized patients, not to use antibiotics, not to use hydroxychloroquine, not to administer drugs by aerosol. Finally, it was stated that “*There is no hard, irrefutable evidence to date (i.e., from controlled clinical studies) of the efficacy of vitamin supplements and food supplements (e.g., vitamins, including vitamin D, lactoferrin, quercetin), the use of which therefore is not recommended herein*”. Such statements are perplexing, in light of the fact that there is no hard, irrefutable evidence to date (i.e., from controlled clinical studies) of the efficacy of paracetamol, which, on the other hand, is always included in the Ministry guidelines.

In light of our current knowledge, this strategy exposed patients to the risk of progression and worsening during the most sensitive period of the disease. In practice, patients at home were left without any treatment other than paracetamol as a symptomatic drug. Later on (26 April 2021), partly due to the continued insistence of physicians working in the field and a number of preliminary publications [17, 29, 30], these guidelines were changed by also introducing the symptomatic use of NSAIDs, but the above-mentioned “remote monitoring” strategy continued to apply.

The latest guidelines of the Ministry [26] illustrate the use of some more recent drugs such as antivirals and monoclonals. Two different approaches are distinguished, based on the types of patients at low risk or at high risk of complications. For those at “low risk”, the indications for monitoring and symptomatic therapies (paracetamol or NSAIDs) are reiterated. High-risk patients are defined as those who have one or more of these characteristics: age> 65 years and male, or smoking habit, or chronic diseases such as neoplasms, immuno-suppressive states, obesity, cerebrovascular disease, dementia, psychotic disorders, pathologies neurodegenerative, cardiovascular diseases (such as arterial hypertension, atrial fibrillation, heart failure, cardiomyopathies, coronary artery disease), type I and type II diabetes mellitus, chronic renal failure, chronic pulmonary disease (COPD), severe or moderate asthma, cystic fibrosis, pulmonary fibrosis, interstitial diseases, pulmonary hypertension. Patients with these risk factors are candidates for early therapy with monoclonal antibodies or with oral or intravenous antiviral drugs.

Currently in Italy for patients with mild - moderate Covid-19 who do not require hospitalization, different treatments are available based on the administration of monoclonal antibodies (combination casirivimab / imdevibam and the antibody sotro-vimab and the combination bamlanivimab / etesevimab) or antiviral agents (nirma-trelvir / ritonavir, remdesevir, molnupiravir). However, efficacy data on the Omicron variant indicate substantial ineffectiveness of the bamlanivamb / etesevimab and casirivimab / imdevimab combinations, while sotrovimab appears to maintain adequate efficacy. The use of lopinavir / ritonavir or darunavir / ritonavir or cobicistat is not recommended for either the purpose of preventing or treating the infection. The randomized clinical trials published to date all conclude that these pharmacological approaches are ineffective.

The selection of the patient to be treated with monoclonal antibodies or antivirals is entrusted to doctors who have the opportunity to come into contact with patients suffering from recent onset COVID-19 and with mild-moderate symptoms. For both types of treatment, the greatest effectiveness is observed with early administration with respect to the onset of symptoms, possibly within 72 hours. This position of the Ministry, which supports a greater efficacy of monoclonals and antivirals in the first 72 hours of the disease, could contrast with the previous statement that in patients with mild disease, no therapies other than symptomatic ones should be carried out (see also figure 2). In fact, in a progressive disease such as COVID-19 it is undoubtedly difficult to establish the evolution of the disease in a short time, especially in the outpatient context and with telemedicine.

## 4. Criticism of the Ministry guidelines by doctors and patients

Faced with the “therapeutic paralysis” brought about by the ministerial guidelines, the doctors of the group “*Early Home Therapy for COVID-19*” appealed to an administrative court (TAR) to obtain more therapeutic freedom and the court decided in their favor, ruling that doctors have (our translation) “*the right/duty, with legal relevance based on both civil and criminal law, to prescribe the drugs they deem most appropriate, guided by science and conscience, which cannot be restricted in the perspective of an expectation, potentially prejudicial both for the patient and, albeit for different reasons, for the doctors themselves*”. The Regional Administrative Tribunal therefore suspended the effectiveness of the measure issued by AIFA with immediate effect and postponed the discussion of the merits to a later date. However, the disputes between doctors and the Ministry continued for months (see Table 2) until recently, without a scientific collaboration agreement that would have been desirable to solve the problem of the best treatment of COVID-19.

The doctors operating within the group “*Early Home Therapy for COVID-19*” requested, on the basis of their observational clinical experiences, the possibility of carrying out randomized and controlled scientific studies, to definitively confirm the efficacy of their treatments, according to an EBM approach, with the assistance of the Ministry of Health and Universities or Research Institutions since their organisation was ill-equipped to single-handedly perform a prospective, randomized and controlled scientific study. This request, to date, has remained unfulfilled.

Nevertheless, several retrospective observational studies have been produced showing that treatment with NSAIDs, administered at the first symptoms of mild-to-moderate COVID-19, resulted in a significant reduction in the number of hospitalizations, with a clear reduction in healthcare expenditure. The efficacy of NSAIDs has been proven “on the field” by rigorously designed, though not strictly randomized, studies [17, 18]. This last paper reported the results of a cohort study in which patients, at the onset of mild symptoms of COVID-19, were treated at home on the basis of a pathophysiological and pharmacological rationale, which included in particular relatively selective cyclooxygenase-2 inhibitors and, when necessary, corticosteroids, anticoagulants, oxygen therapy and antibiotics. A cohort of 108 patients treated at home by their GPs with the “recommended” treatment, between January 2021 and May 2021, was compared with a cohort of 108 patients of similar age, gender and comorbidities, combined with other treatment programs using paracetamol and other drugs (ClinicalTrials.gov: NCT04854824). The primary outcome was COVID-19-related hospitalization, which occurred in only one patient (0.9%) with the “recommended” treatment compared to as many as 12 patients (11.1%) in the comparison cohort (P = 0.0136). The proposed algorithm reduced the cumulative duration of hospital stays by 85% (from 141 to 19 days), as well as the related costs (from € 60,316 to € 9,058). In conclusion, the adoption of the proposed outpatient treatment algorithm during the early and mild phase of COVID-19 reduced the incidence of hospitalization by approximately 10-fold.

A randomized controlled trial of indomethacin versus paracetamol, added to standard background therapy, in which the main outcome was desaturation to <93% O2 [31]. The results showed that no patients in the indomethacin group had desaturated, while as many as 20 patients in the paracetamol group had de-saturated.

Italian physicians who performed home-based therapies during the pandemic have already produced two retrospective studies. The first [7] chose indomethacin for the early home treatment of 153 patients with mild-to-moderate COVD-19 since the beginning of the pandemic, both for its potent anti-inflammatory action and for its documented anti-viral action against various viruses and, in particular, also in cells in vitro and in vivo (in dogs) against coronavirus [2] and more recently also against SARS-CoV-2 [32, 33]. Our multi-therapeutic treatment is based on a therapeutic rationale combining various molecules with anti-inflammatory, antiviral and antioxidant properties, such as indomethacin, hesperidin, quercetin, aspirin in an antiplatelet dosage, and gastric protection with omeprazole [34]. Intervention within 72 hours resulted in zero hospitalizations with a reduction in the duration of symptoms and a clear improvement in disease outcomes, compared to what was observed in a comparison group of patients who had waited more than 72 hours before calling the doctor.

Another retrospective observational study, recently released in preliminary form [8] investigated the characteristics, management and outcomes in COVID-19 patients treated in Italy by 10 volunteer physicians within the *IppocrateOrg* Association, between 1 November 2020 and 31 March 2021. 392 consecutive COVID-19 patients with various types of comorbidities were treated with vitamins and supplements (98.7%), aspirin (66.1%), antibiotics (62%), glucocorticoids (41.8%), hydroxychloroquine (29.6%), enoxaparin (28.6%), colchicine (8.9%), oxygen therapy (6.9%), and ivermectin (2.8%). Admission occurred in 5.8% of the total cases, 390 patients (99.6%) recovered, one patient (0.2%) was lost at follow-up and one patient (0.2%) died after hospital admission. It should be noted that the COVID-19 lethality in our cohort was 0.2% (95% confidence interval: 0.01–1.4%), whereas the overall COVID-19 lethality in Italy over the same period was between 3% and 3.8%. The use of the single drugs and drug combinations described in this study therefore appears to be effective and safe, as indicated by the few mild adverse reactions reported.

These results are prompting calls for a study with a non-inferiority protocol between the different therapeutic schemes and any of the antiviral drugs already authorized in the early treatment of COVID-19. In the event of a positive non-inferiority result of our therapeutic scheme against the chosen antiviral, there would be a truly significant reduction in therapeutic expenditure.

## 5. Paracetamol: not as safe as was thought?

Paracetamol [N-(4-hydroxyphenyl) acetamide, N-(4-hydroxyphenyl) ethanamide] is a drug with analgesic and antipyretic action, widely used in common over-the-counter preparations for viral colds, or in drugs for the treatment of acute and chronic pain. It is also among the most widely used drugs in the home treatment of COVID-19, also because it has been recommended by the Ministry of Health since the first guidelines were issued. However, doubts about the validity of its use soon emerged [35], based on the fact that it could interfere with glutathione reserves in the body. In fact, when administered in high doses, this drug is metabolized with the formation of N-acetyl-p-benzoquinone imine (NAPQI), which in turn requires glutathione for hepatic catabolism, to the point that that the antidote for paracetamol intoxication is N-acetylcysteine. Glutathione is an indispensable element of cell metabolism and, in particular, of the defense against toxic oxygen derivatives (ROS) and thus cellular oxidative stress [35–38]. Furthermore, NAPQI metabolism in GSH deficiency leads to an accumulation of denatured proteins by alteration of their sulfhydryl residues.

Oxidative stress has been observed in infection by such viruses as hepatitis B [39], hepatitis C [40], influenza [41] and SARS-CoV-2 [42, 43]. In the latter case, excess ROS could also lead to an unfavorable evolution in elderly subjects with low antioxidant capacity [44, 45], possibly because the intracellular redox environment alters antigen presentation [46] and the expression of ACE2 [47, 48]. Indeed, the severity and mortality risk of SARS-CoV-2 or COVID-19 have been associated with age [45]. It seems possible that the oxidation of thiols (-SH residues of proteins) into disulfides (S-S), under an oxidative stress mechanism, increases the affinity of spike proteins for the ACE2 receptor and thus increases the severity of COVID-19 [47].

It therefore seems appropriate to reassess the risk-benefit ratio of paracetamol in viral infections [49] particularly in patients with reduced liver function. Indeed, 37.2 %–76.3 % of patients with COVID-19 presented abnormal liver function during COVID-19 [50]. Furthermore, in a retrospective cross-sectional study conducted in 4 public hospitals in Peru, in which 1,100 patients admitted with a diagnosis of COVID-19 were enrolled [51], it was found that liver enzymes (AST and ALT) were altered on admission in more than 60 per cent of patients. Moreover, patients taking paracetamol featured a higher risk of liver damage: OR=1.41 (95% CI: 1.01–1.98; p=0.04). Worsening prognostic factors were dyslipidemia and fever; improving prognostic factors were the use of corticosteroids, enoxaparin and the female gender.

N-acetylcysteine, which supports glutathione and thus the main antioxidant defense systems [52] has been used with good results in influenza syndromes [53] and acute respiratory distress syndrome (ARDS) [54] and has been suggested as a potential therapeutic agent for COVID-19 [37, 55–58]. Some positive results have been obtained in critically ill patients [59, 60], although a randomized study conducted in Brazil on a patient group treated with an infusion of 21 grams of N-acetylcysteine/day, obtained no therapeutic effect [61]. Further studies are needed to better evaluate effective dosages and application at various stages of the disease. Acetylcysteine was also used to counteract remdesivir-induced liver failure in two COVID-19 patients [62].

In conclusion, paracetamol, which lacks anti-inflammatory activity and risks depleting the glutathione that is necessary for antiviral defense purposes, does not appear to be the drug of choice for the treatment of patients with COVID-19 at an early stage.

## 6. Discussion

The problem presented here is part of the continuous debate between EBM, based on randomized trials and meta-analyses, and the doctor’s freedom of decision, based on observational studies, personal experience and often intuition, also taking into account the individual characteristics of each patient. Furthermore, the experience of Italian doctors engaged on the front line in the battle against the COVID-19 disease suggests that the “guidelines” or the “recommendations” of the public health authorities, governed by political trends, risk blocking the search for new therapeutic approaches, if they are intended to be followed in a too rigid and indisputable way. Paradoxically, the need to formulate guidelines based on rigorous evidence of efficacy can become an obstacle to the exploration of new therapies in pandemic emergency conditions.

However, this apparent contrast is based on a misconception of EBM. One of the best definitions of EBM is the following “*Evidence based medicine is the conscientious, explicit, and judicious use of current best evidence in making decisions about care of individual patients. The practice of evidence-based medicine means integrating individual clinical expertise with the best available external clinical evidence from systematic research*” [63]. Therefore, during a little-known virus pandemic, it would have been very useful to integrate the scant indications from EBM with the individual clinical experience that many doctors had gained by curing promptly at home patients with COVID-19.

According to the decision by the Council of State of 9/02/2022 No. 00411/2022 REG.RIC, the ministerial circular “*does not ban or restrict the use of drugs but merely lays down recommendations and guidelines for the various treatments, based on the best available evidence in the literature, depending on the occurrence of specific conditions*”. The decision then stated that the circular “advises against” – but does not explicitly prohibit – the use of certain drugs in the home treatment of Covid-19. Individual doctors are free to exercise their professional judgment, under their own responsibility, when prescribing the drugs they deem most appropriate in each specific case, in relation to the individual patient, on the basis of the scientific evidence collected. This “open” perspective is certainly acceptable, although the decision then goes on to state that “*Doctors cannot prescribe a drug based simply on intuition or improvisation experimented on the individual patients, but on scientific evidence and therefore on rigorous studies and precise clinical trials, now numerous at the international level even in the fight against the Sars-Cov-2 virus, after two years since the beginning of the pandemic*.”

Therefore, the general question remains open as to the level of “evidence” required in the face of a new and complex disease, which affects patients with several comorbidities, and goes through several stages. The adoption of guidelines, especially in the medical sphere, has already given rise in the past to extensive discussions on their legal nature and classification within the framework of legal sources. On the one hand, their inherent nature as guidelines would prevent them from being considered in regulatory terms, given that they are indicative and do not claim to be exhaustive; on the other hand, some have argued that the wide use of these instruments give them a “binding force” in an emergency [24].

These issues end up influencing the operations of physicians working in contact with patients in emergency conditions, and awareness of this problem has led various groups of physicians to call for greater attention to be paid to their experiences, even in the absence of definite indications from EBM. Particular mention should be made of a technical document prepared by a group of experts and submitted to the attention of Italian political forces in the spring of 2021 [64]. This paper clearly illustrates the situation in which doctors found themselves working at that time.

The doctors claimed that “*Since no study has so far been proposed, conducted or published on the home therapy of patients with COVID-19, a doctor faced with a new and complex condition such as the one in question – taking into account the available scientific evidence, biological plausibility and the patient’s specific situation – may adopt the therapeutic approaches that he or she deems necessary guided by science and conscience*”. Furthermore, “*doctors should be given the possibility of proposing on- and off-label drugs to patients, as well as useful indications for the prevention and treatment of diseases that can complicate the course of COVID-19, also by way of general evidence-based nutritional education and specific advice on nutrition (also to ensure a proper intake of vitamins and flavonoids, without ruling out the use of supplements where necessary*”. Among the drugs that may prove useful, according to the doctor’s judgement in individual cases, for the prompt home management of patients with COVID-19, are NSAIDS and, in particular, indomethacin, that in addition to the anti-inflammatory and anti-bradykinin actions, also demonstrated a clear antiviral action in vitro and in vivo against SARS-CoV-2 [2, 7, 31, 32, 34, 65]. While a recent meta-analysis, performed by analyzing 40 studies, showed that the use of NSAIDs did not reduce mortality outcomes among people with COVID-19, it has also shown that NSAIDs can be used safely among patients positive to SARS-CoV-2 [66]. Moreover, it should be noted that the latter analysis assessed the effectiveness of ibuprofen, aspirin and COX-2 inhibitors, it did not mention studies with indomethacin. The conclusions express the hope that the therapeutic experience built up in the home set-ting, about with regard to some of the indicated drugs and their combinations, or even others that might prove useful in the treatment of COVID-19 patients over time, would be the subject of highly valid studies that could evaluate them and allow appropriate recommendations for their use.

As a result of this work, the political forces decided to commit the government (a) to modifying the guidelines, which were considered too restrictive, and (b) to setting up a ministerial monitoring committee made up of representatives of all the professions involved in local community health care. Other proposed measures included implementing interventions from the diagnosis stage capable of involving all health care, social service and family support personnel to ensure that the various experiences and clinical data collected by the regional health systems flowed into a national protocol for the home management of Covid-19 patients, and, last but not least, to support this protocol with a plan to increase the supply of telemedicine devices suited to ensuring adequate and constant monitoring of the patients’ clinical condition [64]. Unfortunately, to this day, the guidelines have remained basically unchanged (the only difference being the addition of NSAIDs, alongside paracetamol, among the symptomatic drugs) and none of these objectives proposed to the government by the political forces has been implemented.

From the point of view of the official regulations, the situation is entirely fluid and no precise definition of physicians’ rights and duties, as well as the extent of “therapeutic freedom” possible in conditions of uncertainty, has yet been agreed on. The Council of State’s ruling on the AIFA Guidelines for the Home Management of Patients Infected with Covid-19 and the Ministry of Health’s circular “*Home Management of Patients Infected with SARS-CoV-2*”, updated on 26 April 2021, contain mere recommendations and not binding requirements and are, in legal terms, merely guidelines for general practitioners, as benchmarks for current experiences in therapeutic methods at an international level.

Evidence of a pathological link between metabolic diseases and severe forms of COVID-19 stimulates critical reflection and new considerations. Precisely because COVID-19 is a disease that most severely affects people with poor health, diabetes, hypertension and metabolic syndrome, correct supplementation with dietary factors may be the key to preventing and countering the complications of COVID-19. It is highly plausible that a set of natural agents can inhibit the cytokine storm and hypercoagulability that characterize a severe COVID-19 infection: vitamin D3, omega-3 polyunsaturated fatty acids, other nutraceuticals that can activate anti-inflammatory and antioxidant pathways such as quercetin, rutin, vitamin C, zinc, melatonin, lactoferrin and glutathione [34, 67–71]. Despite the absence of meta-analyses, there is much preliminary evidence, even in double blind, that quercetin can have positive effects on the evolution of the disease, possibly also in association with antivirals [72–76].

Each patient should be evaluated for his/her individual susceptibility and for this reason the evidence deriving from controlled clinical trials (which involve administration in homogeneous groups and under standardized conditions) are important but do not exhaust the possibilities of medical intervention. From this point of view, it can be understood that the guidelines cannot yet systematically “recommend” these innovative approaches, but equally it would be reductive and counterproductive if they were to ban them. Rigorous studies will be fundamental to validate preventive and therapeutic protocols that could combine supplements, vitamins and antioxidants with chemical drugs in aid protocols to mitigate disease progression following SARS-CoV-2 infection.

## 7. Conclusions and Perspectives

The Italian approach to the pandemic by the institutions has revealed significant shortcomings and has proved to be partly mistaken, based on the contents of this paper, with the most prominent mistakes, in our opinion, being: the decision to tackle the pandemic only through the development of vaccines based on novel technology, compared to the past, and therefore with insufficient information as to the duration of their efficacy and their medium and long-term safety; the issuing of guidelines that discouraged doctors from adopting early home treatment therapy guided by science and conscience, using the drugs considered most appropriate for each patient; the decision to strongly recommend “*watchful waiting*” and symptomatic drugs alone, especially paracetamol, going so far as to sanction doctors who did not comply with the official recommendations; the refusal to enter into any form of dialogue with doctors who promptly treated thousands and thousands of patients at home, enormously reducing the number of hospitalizations. This inefficient management of the pandemic can be viewed as one of the factors whereby Italy is among the countries with most hospitalizations and deaths due to COVID-19.

Looking ahead, it is necessary to adopt a much more open and flexible approach, starting systematic and comparative studies between the different protocols that emerged from the experience of doctors who work at the patient’s bedside or through telemedicine in the early stages of the disease. Since vaccine prevention and early treatment are not alternatives, it would be necessary, in the near future, for resources to be allocated to systematic and patient research of the various therapeutic approaches that have shown promising results, without focusing only on antiviral drugs in the belief that they can constitute a “silver bullet” capable of curing the disease in all instances. Relying only on “big pharma”, which is engaged in researching next-generation antivirals, could prove yet another serious mistake in the face of a disease as complex as COVID-19.

## Figures and Tables

**Table 1:** Lethality during the pandemic in Italy and Portugal compared (from Worldometers Coronavirus site, https://www.worldometers.info/coronavirus/).

	Italy			Portugal		
Year	2020	2021	2022*	2020	2021	2022*
**No. Cases**	2,000,000	4,000,000	10,700,000	420,000	1,412,000	2,412,000
**No. Deaths**	70,000	62,300	29,500	6,197	12,083	3631
**Lethality**	3.5	1.55	0.32	1.64	0.85	0.15

* Until 18 of May 2022

**Table 2:** Timeline of the key official actions concerning home-based care in Italy during the COVID-19 pandemic

Date	Actor	Actions concerning home treatment	References
22-Jul-20	Italian Medicine Agency (AIFA)	The use of hydroxychloroquine, alone or in combination with other drugs, outside of clinical trials, was suspended.	Note of AIFA https://www.aifa.gov.it/documents/20142/1123276/idrossiclorochina_22.07.2020.pdf Accessed 13 June 2022
30-Nov-20	Italian Ministry of Health (Ministry)	Issued a circular entitled “*Home management of patients with SARS-CoV-2 infection*”.	Circular 0024970-30/11/2020-DGPROGS-DGPROGS-P [3]
10-Dec-20	Italian Council of State	The note of 22 July 2020 from AIFA, which prohibited the off-label prescription of hydroxychloroquine, was suspended	Judgement n. N. 09070/2020 REG.RIC. allegato7506782.pdf (quotidianosanita.it)
4-Mar-21	Regional Administrative Tribunal (TAR) of Lazio, Rome Branch, div. III	At the request of a doctors’ and patients’ association, the Ministry’s circular with guidelines for the home management of patients was cancelled	TAR Lazio, Div. III *quater,* precautionary order no. 1412.
8-Apr-21	Italian Senate	Approved an agenda with a commitment by the government to update the protocols and guidelines for the home care of Covid-19 patients, taking into account all the experiences of professionals working in the field.	At the end of the session held on 7 April 2021 https://www.senato.it/3818?seduta_assemblea=19801
26-Apr-21	Ministry	Updated the outpatient therapy guidelines with the addition of symptomatic use of anti-inflammatory drugs, while maintaining the recommendation of paracetamol	Circular 0017948-26/04/2021-DGPRE-MDS-P [25]
15-Jan-22	Lazio TAR (Rome, div. III)	At the request of a doctors’ and patients’ association, cancelled the new guidelines for the home management of patients, as contrary to the professional and ethical standards of doctors	Judgment No. 419/2022
19-Jan-22	Council of State	Cancelled the 15 January ruling of the Lazio TAR	Judgement of 9 February 2022, no. 946. https://www.quotidianosanita.it/governo-e-parlamento/articolo.php?articolo_id=102213
10-Feb-22	Ministry	Updated the guidelines by replacing “*watchful waiting*” with “*monitoring*”, while maintaining the recommendation of symptomatic use of paracetamol or NSAIDs	Circular 0003435-10/02/2022-DGPROGS-MDS-P [26]
